# Alkyl Xylosides: Physico-Chemical Properties and Influence on Environmental Bacteria Cells

**DOI:** 10.1007/s11743-017-2012-2

**Published:** 2017-09-01

**Authors:** Wojciech Smułek, Ewa Kaczorek, Zuzana Hricovíniová

**Affiliations:** 1 Institute of Chemical Technology and Engineering, Poznan University of Technology, Berdychowo 4, 60-965 Poznan, Poland; 2 Institute of Chemistry, Slovak Academy of Sciences, Dúbravská cesta 9, 845 38 Bratislava, Slovakia

**Keywords:** Foaming properties, Microbiology, Non-ionic surfactants, Surface activity

## Abstract

A group of four selected non-ionic surfactants based on carbohydrates, namely octyl d-xyloside (C_8_X), nonyl d-xyloside (C_9_X), decyl d-xyloside (C_10_X) and dodecyl d-xyloside (C_12_X), have been investigated to accomplish a better understanding of their physico-chemical properties as well as biological activities. The surface-active properties, such as critical micelle concentration (CMC), emulsion and foam stability, the impact of the compounds on cell surface hydrophobicity and cell membrane permeability together with their toxicity on the selected bacterial strains have been determined as well. The studied group of surfactants showed high surface-active properties allowing a decrease in the surface tension to values below 25 mN m^−1^ for dodecyl d-xyloside at the CMC. The investigated compounds did not have any toxic influence on two *Pseudomonas* bacterial strains at concentrations below 25 mg L^−1^. The studied long-chain alkyl xylosides influenced both the cell inner membrane permeability and the cell surface hydrophobicity. Furthermore, the alkyl chain length, as well as the surfactant concentration, had a significant impact on the modifications of the cell surface properties. The tested non-ionic surfactants exhibited strong surface-active properties accompanied by the significant influence on growth and properties of *Pseudomonas* bacteria cells.

## Introduction

Surfactants of natural origin are gaining ever-growing interest because of their great potential driven by a strong demand for biodegradable products. They have attracted increasing attention as they are ecologically suitable alternatives to their synthetic counterparts derived from petrochemical resources. Sugar-based amphiphiles—alkyl glycosides are intensively studied compounds due to their widespread applications in various fields [1, 2].

Long-chain alkyl glycosides belong to the group of non-ionic surfactants. These amphiphilic compounds contain hydrophilic and hydrophobic regions of the molecule, which allow them to accumulate between fluid phases and reduce the surface and interfacial tensions as well [3]. Besides their excellent surfactant properties, their low toxicity and good biodegradability are important advantages. Moreover, they can be produced from renewable sources, which makes them environmentally friendly chemicals. Additionally, their synthesis fulfills requirements of green chemistry principles, such as the use of safe solvents and substrates and high energy efficiency [4]. Moreover, the alkyl derivatives of carbohydrates can enable formulation of stable and useful emulsions and microemulsions [5]. Hence, the alkyl derivatives of saccharides have a variety of uses in the food industry and in the production of cosmetics and detergents [6, 7]. Mildness and protection for the skin as well as high-quality standards by minimization of by-products and trace impurities make the alkyl polyglucosides preferred components of personal care products [5]. The utilization of surfactants from natural sources in the medical field has increased significantly during the past decade [2]. The alkyl derivatives of carbohydrates are recognized as valuable parts of drug-delivery systems, which generate no harmful derivatives in the human organism [8]. Additionally, these surfactants are considered to be very promising agents in bioremediation, with recent examples in surfactant-enhanced biodegradation of hydrophobic pollutants [9], phytoremediation of polycyclic aromatic hydrocarbons [10] and washing of crude oil contaminated soils [11].

Hemicelluloses are the second most abundant polysaccharides occurring in nature. These essentially linear polymers with short-branched chains can be easily hydrolyzed to monomeric sugars. One of the main components of hemicelluloses is xylan. Its molecule has a backbone with 1,4-linked β-d-xylopyranose units (xylose), hence can be an efficient source of this monosaccharide [12].

The methods of synthesis of alkyl derivatives of xylose include enzymatic reactions [12, 13] as well as a one-pot process with sulfuric acid as catalyst [14]. Sekine *et al*. [15] presented their synthesis using ionic liquids. Additionally, Ludot *et al*. [16] used sulfoxides and sulfones as solvents in a non-catalyst process. Recently, Hricovíniová has described a microwave-assisted, transition-metal catalyzed glycosylation for the preparation of various amphiphilic alkyl glycosides [17, 18].

The physical and chemical properties of the surfactants depend strongly on the alkyl chain length. The critical micelle concentration (CMC) of cationic surfactants is proportional to the hydrophobic chain length and varies with the carbon number in the alkyl chain [19]. Additionally, with an increasing number of carbon atoms in the alkyl chain, the surface area occupied by the surfactant molecule on interphase surface increases [20]. Moreover, the alkyl chain length of a surfactant can affect its foaming properties [21]. The lengthening of the alkyl-chain enhances the surface activity of zwitterionic imidazolium-based surfactants [22]. The interfacial dilational properties of several hydroxy-substituted alkyl benzenesulfonates with different alkyl chain lengths were tested by Wu *et al*. [23]. The correlation between alkyl chain lengths and the surface active properties was also described in the case of choline carboxylate soaps [24], sodium-*N*-acyl sarcosinate (SNAS) and *N*-cetylpyridinium chloride (CPC) surfactants [25] as well as sophorolipids produced by *Candida bombicola* [26].

The influence of alkyl chain length on surfactant properties also refers to alkyl derivatives of carbohydrates. The change of the surface properties depends on the alkyl length and the hydrophilic carbohydrate headgroup size among *n*-alkyl-*O*-melibiosides and *n*-alkyl-*O*-cellobiosides [27]. Similarly, the properties of alkyl maltosides and glucosides have also been described in the literature [28, 29]. For example, the wide review of alkyl glucosides properties and applications was prepared by von Rybinski and Hill [5].

Thinking about an extensive use of surfactants, their influence on ecosystems should be investigated. Surfactants are ubiquitous pollutants present in wastewaters and soils [30]. Hence, their impact on the environment is of great interest [31]. One of the research areas is the influence of surfactants on environmental bacteria. Apart from direct toxicity on microorganisms, surface active agents can modify cell membrane permeability [32], zeta potential of cell surfaces [9] and bacterial adhesion to organic or solid phases [33, 34]. The environmental effect of surfactants is also connected with their alkyl chain length [35]. It also refers to cell surface properties [36].

The aim of the research was the investigation of physical and chemical properties and the evaluation of the structure–property relationship of four alkyl d-xylosides varying in chain length. The dependency between surface tension and surfactant concentration was analyzed in order to evaluate the critical micelle concentration (CMC) of alkyl xylosides, as well as their emulsification properties and foam stability. The second part of the research was aimed at identifying the impact of the studied compounds on bacterial cells from the *Pseudomonas* genus, found frequently in the environment. The toxicity effect of the surfactants was determined as well as changes of cell surface hydrophobicity and cell membrane permeability. The study will provide a broad insight into surface active properties of alkyl xylosides. Moreover, it will allow an assessment of the potential environmental risk related to with their contact with microorganisms.

## Materials and Methods

### Chemicals

All fine chemicals employed in this study were of highest purity grade, produced by Sigma-Aldrich (Germany). Conversions, purities and structure of products were determined by NMR spectroscopy. High-resolution NMR spectra were recorded in a 5 mm cryoprobe on a Bruker Avance III HD spectrometer at 14T. The experiments were carried out at 25 °C in chloroform-d_6_ (CDCl_3_). The proton and carbon chemical shifts were referenced to internal TMS. One-dimensional 600 MHz ^1^H- and 150 MHz ^13^C-NMR spectra, as well as two-dimensional COSY, HSQC and HMBC, were used for determination of ^1^H- and ^13^C-chemical shifts. Microwave reactions were performed in a multimode microwave reactor, CEM Discover, consisting of a continuously focused microwave power delivery system with operator-selectable power 0–300 W, and microwave frequency source of 2.45 GHz.

### Synthesis of Alkyl Xylosides

**Fig. 1:**
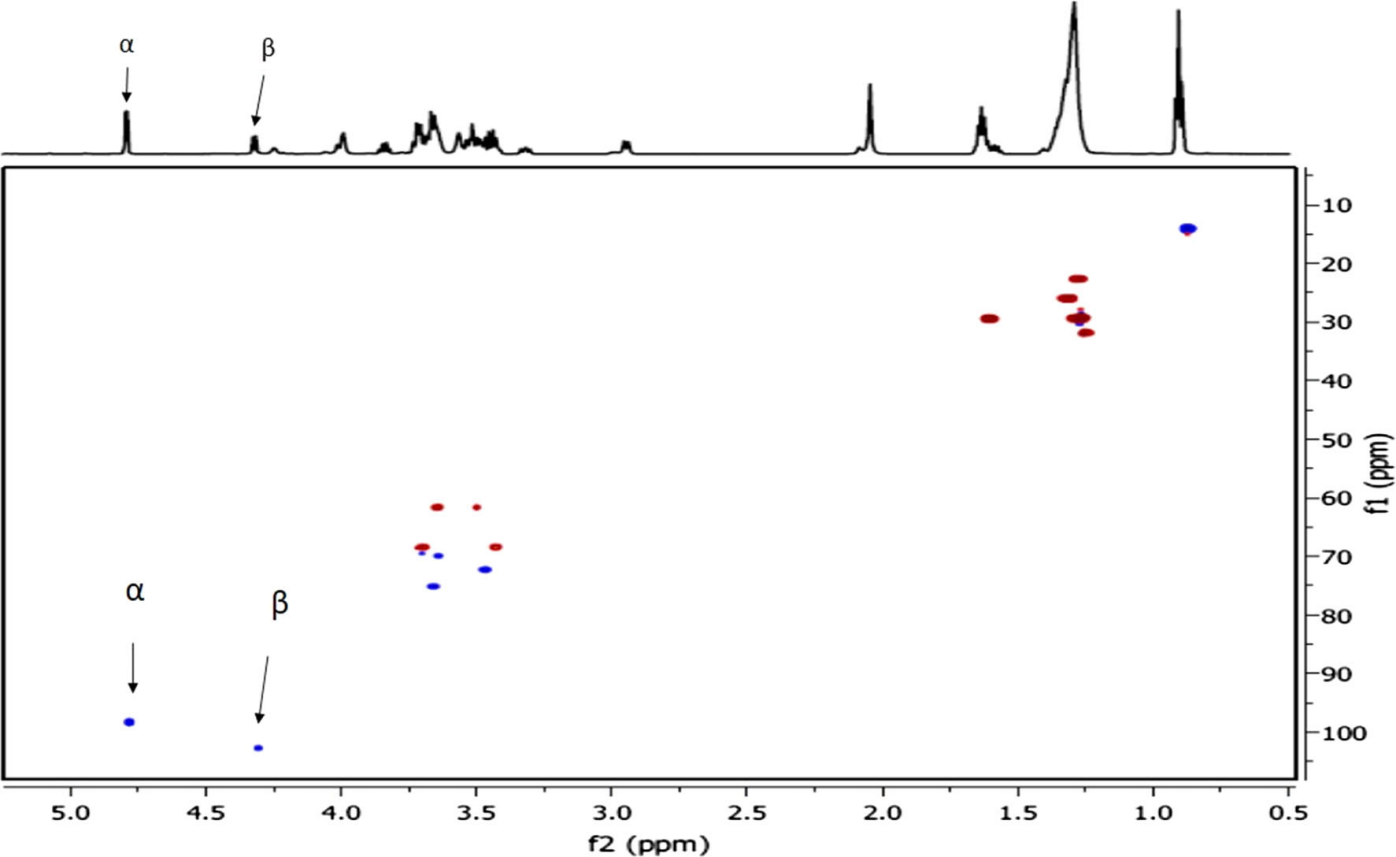
2D HSQC spectrum and ^1^H-NMR conventional spectrum of 1-nonyl-α/β-d xylopyranoside, collected under the same experimental conditions

Monomeric d-xylose was obtained after microwave-induced Mo(VI)-catalyzed hydrolysis of beechwood xylan. The analysis of ^1^H-NMR spectra indicated that xylan was completely hydrolyzed and converted to the equilibrium mixture of pentoses (d-xylose and d-lyxose) as described previously [37]. A homologous series of amphiphilic long-chain alkyl d-xylosides [octyl d-xyloside (C_8_X), nonyl d-xyloside (C_9_X), decyl d-xyloside (C_10_X), and dodecyl d-xyloside (C_12_X)] were prepared by microwave-assisted synthesis by direct coupling of unprotected d-xylose and the corresponding long-chain alcohol, in the presence of phosphomolybdic acid (PMoA) as promoter [18]. d-Xylose (6.66 mmol, 1 equiv.), PMoA/SiO_2_ (0.06 mmol) and 5 equiv. of corresponding alcohol (C_8_–C_12_) were mixed in Pyrex glass tubes and sealed with Teflon septa. The reaction mixture was exposed to microwave radiation (150 W) for 1–10 min. Work-up of the reaction mixture afforded the crude products which were purified by flash column chromatography on silica gel. The ratio of α/β anomers for individual alkyl d-xylosides present in the thermodynamic equilibrium mixture was determined by ^1^H-NMR spectroscopy. For the long-chain alkyl d-xylosides, the α-anomers were more prevalent; the anomeric ratio ranged from 57:43 (α:β) for C_8_X, 61:39 for C_9_X, 60:40 for C_10_X and 68:32 for C_12_X. Magnitudes of proton–proton spin–spin coupling constants across three bonds between H-1 and H-2 protons (^3^ *J*_H1–H2_) were 3.6–3.8 Hz for C_8_X–C_12_X for α-anomers. The ^3^ *J*_H1–H2_ magnitudes agree with the ^4^C_1_ form of the pyranose ring for these derivatives. The representative a 2D HSQC spectrum and a ^1^H-NMR conventional spectrum of 1-nonyl-α/β-d xylopyranoside, collected at the same experimental conditions, are shown in Fig. 1.

### Surface Active Properties

#### Surface Tension Measurements

In order to measure the equilibrium surface tension of the surfactants' water solutions the du Noüy ring technique with an Easy Dyne K20 tensiometer with a platinum ring (Krüss, Germany) was used. The experiments were performed at 22 ± 1 °C. The surface tension data were fitted by adsorption equations. From a physicochemical point of view, the most appropriate was found to be the Szyszkowski equation [36]. Additionally, the solubility of the surfactants in deionized water was also evaluated [38].

#### Emulsification Test

The emulsification properties were evaluated based on measurements of optical density of the emulsion. The higher optical density is, the more dispersed the organic phase is [39]. 2.5 mL of the surfactants solutions in deionized water (18.2 MΩ cm) at concentrations 10, 50, 100 and 250 mg L^−1^ were added to glass tubes. The sample was supplemented with 25 µL of hexadecane. After mixing (using vortex shaker) for 1 min, the sample was transferred into a spectrophotometric cuvette. The optical density at 600 nm (OD_600_) was measured for the first time immediately after shaking, and again after 15 min.

#### Foam Stability Test

The foam stability test was conducted according to the method described by Belhaij and Mahdy [40]. The foam was generated during 1 min in a glass tube (2 cm diameter) filled with 20 mL of surfactant solution, prepared as in the emulsification test. The height of the foam layer was measured at the moment the foam generation was stopped and 5 min after that moment.

### Microorganisms and Growth Conditions

Further experiments were carried out using two bacterial strains from *Pseudomonas* genera: a reference strain *Pseudomonas fluorescens* ATCC 14700, and a strain *Pseudomonas* sp. KG1 (GenBank number KP096515), which was isolated from soil contaminated with crude oil, collected from sites in Southern Poland. The strains were kept on plates with Mueller–Hinton agar. The composition of culture mineral salts medium used throughout these studies was described by Kaczorek *et al*. [41]. A liquid culture was started by adding a loopful of cells from an agar plate into a 250 mL Schott Duran^®^ laboratory glass bottles containing 50 mL of medium, and incubated overnight. Then, 5 mL of this liquid culture was used for the inoculation of the final culture to reach an optical density (measured at 600 nm) ca. 1.0.

### Toxicity Measurements

The toxicity of the tested surfactants on used bacterial strains was determined using the MTT cell proliferation assay, which is based on a transformation of tetrazolium dye MTT (3-(4,5-dimethylthiazol-2-yl)-2,5-diphenyltetrazolium bromide) to its formazan. This reaction is catalyzed by cellular reductases. The activity of these enzymes can be used for evaluation of the cell viability. The method was conducted according to Jafarirad *et al*. [42] after some modifications. Briefly, in 1.5 mL Eppendorf tubes the 0.1 mL of the MTT solution (5 g L^−1^) and 0.5 mL of bacteria cells suspension (OD_600_ = 1.0) in the culture mineral medium were mixed. The samples were supplemented with the surfactants stock solutions. Finally, the different surfactant concentrations in the samples (25, 100, 250, 500, 1000 mg L^−1^) were obtained. All samples were quenched up to 1.0 mL with the culture mineral medium. The control was the sample without the surfactant addition. Then the samples were incubated (24 h, 30 °C) and shaken gently. Thereafter, the samples were centrifuged (15,000*g* for 5 min), and after supernatant removal, the precipitate was dissolved in 1 mL of propan-1-ol. The samples were vortexed and centrifuged (15,000*g*, 5 min) again. Then, the absorbance of the supernatant at 560 nm was measured (V-650 UV–Visible Spectrofotometer, Jasco, Japan). All values obtained were referred to the value obtained for the control.

### Cell Inner Membrane Permeability

In order to evaluate the changes of the inner membrane permeability, the modified method described by Zhang *et al*. [43] was applied. Bacteria cells from the exponential growth phase were centrifuged at 8000*g* for 5 min and cell suspension in a culture mineral medium was prepared. Then 0.1 mL of *o*-nitrophenyl-*β*-d-galactopyranoside (ONPG) in concentration 30 mmol L^−1^ and 0.5 mL of bacteria cells suspension (OD_600_ = 1.0) in the culture mineral medium were added to 1.5-mL Eppendorf tubes. The samples were supplemented with the surfactant stock solutions to obtain the different surfactant concentrations in the samples (10, 25, 100, 250, 500, 1000 mg L^−1^). All samples were quenched up to 1.0 mL with the culture mineral medium. The control was the sample without the surfactant addition. Next, the samples were incubated for 2 h at 28 °C. After this time suspension was centrifuged at 4000*g* for 5 min and the supernatant was determined to be under 415 nm.

### Cell Surface Hydrophobicity

To determine the cell surface hydrophobicity the microbial adhesion to hydrocarbon method [44] in a modified version was chosen [45]. In prepared bacterial cultures the tested alkyl xylosides (at different concentrations 10, 25, 50 and 100 mg L^−1^) were used as carbon and energy sources. The control sample, without any surfactant, was the culture with glucose (200 mg L^−1^). The measurements were conducted after 7 days of incubation.

### Statistical Analysis

All experiments were conducted in triplicate, and the mean values and statistical error were calculated. All the results obtained were statistically analyzed using SigmaStat 11.0 software.

## Results and Discussion

### Surface Active Properties

The dependency between the surface tension and concentrations of the surfactants solutions was fitted to the Szyszkowski equation of the adsorption isotherm and the adsorption parameters were calculated (Table 1). These parameters allow a description of the behavior of the surfactant molecules in interphase. Considering the Gibbs free energy of adsorption, there can be a noticeable increase of it with a rising number of carbon atoms in alkyl chain. However, the values of the minimum surface occupied by a surfactant molecule are not correlated with alkyl chain length. The lowest minimum surface occupied by single surfactant molecule was obtained for C_12_X (2.69 × 10^−19^ m^2^), when the highest value of this parameter was noticed for C_10_X (4.68 × 10^−19^ m^2^). This phenomena could be interpreted that an alkyl chain length increase of two carbon atoms from decyl to dodecyl involves a significant change of molecular position in the interphase. The water solubility of the tested compounds was evaluated as 1.0, 0.42, 0.20 and 0.13 g L^−1^ for C_8_X, C_9_X, C_10_X and C_12_X, respectively. Below these concentrations, the surfactants’ solutions were colorless and transparent.

**Table 1:** The evaluated adsorption parameters from the Szyszkowski equation for tested alkyl d-xylosides

Surfactant	*Γ*_*∞*_ [mol m^−2^]	*A*_min_ [m^2^]	−Δ*G*_ads_ [kJ mol^−1^]
Octyl d-xyloside C_8_X	4.27 × 10^6^	3.89 × 10^−19^	23.2
Nonyl d-xyloside C_9_X	4.61 × 10^6^	3.61 × 10^−19^	24.8
Decyl d-xyloside C_10_X	3.55 × 10^6^	4.68 × 10^−19^	30.6
Dodecyl d-xyloside C_12_X	6.18 × 10^6^	2.69 × 10^−19^	32.0

*Γ*_*∞*_, surface excess at the saturated interface; *A*_min_, minimum surface area occupied by statistical molecule; −Δ*G*_ads_, Gibbs free energy of adsorption

The critical micelle concentrations (CMC) for C_9_X (3.010 mM), C_10_X (0.689 mM) and C_12_X (0.049 mM) were also evaluated. As mentioned above, the maximum solubility of C_8_X in water was observed at the concentration 1.0 g L^−1^ (3.8 mM). However, for surfactants from one homologous series there is a direct and simple correlation between the length of hydrocarbon chain and the logarithm of the CMC [19]. This dependency is also observed for the tested surfactants. Finally, the correlation equation was found and it allowed the calculation of the CMC for C_8_X (11.3 mM).

The surface active properties of alkyl xylosides were discussed by Bouxin *et al*. [46]. They found the CMC of octyl and decyl d-xylosides as 953 mg L^−1^ (3.63 mM) and 301 mg L^−1^ (1.03 mM), respectively. The presented results are three times lower than the values presented in these research, but the differences can be explained by different methods and conditions of the surface tension measurements. Additionally, the authors also used other synthesis method to obtain alkyl xylosides than in the presented study. Moreover, Marinkovic *et al*. [47], who used wheat straw as a substrate in the synthesis of decyl xylosides, observed the higher CMC values of obtained product than in the case of the surfactant produced from the pure d-xylose. The experimental data indicate the important influence of purity of the substrate on product properties.

The CMC of different alkyl derivatives of carbohydrates have been presented in several publications. López *et al*. [48] studied alkyl glucosides properties and presented the CMC values for octyl, nonyl, decyl and dodecyl glucoside: 18.0, 5.6, 1.8, 0.18 mM, respectively. Chaveriat *et al*. [49] observed the surface tension 28 mN m^−1^ at the CMC (12 mM) for 6-*O*-octyl-d-galactopyranose. Zhang *et al*. [50] found that the CMC value of *n*-dodecyl-ß-d-maltoside equals to 0.18 mM.

Moreover, Laurent *et al*. [51] tested a different group of sugar-based surfactants, glucuronamides, and observed for octyl and dodecyl derivatives the CMC at 3.3 mM (*γ*_CMC_ = 24.4 mN m^−1^) and 0.7 mM (*γ*_CMC_ = 22.1 mN m^−1^) respectively. To illustrate, the CMC values for commercial surfactants are as followed: 0.08 mM for Tween^®^ 20 [52], 20 mM for Hecameg^®^ [52], 8.35 mM for sodium dodecyl sulfate [53], 15 mM for dodecyltrimethylammonium bromide [50].

The tested compounds showed very strong surface active properties. The CMC values for alkyl xylosides were several times lower than those obtained for glucosides with analogical alkyl chains [48]. Simultaneously, the alkyl xylosides presented a significant possibility for a surface tension decrease. However, the surface tension values at CMC were comparable with those for glucuronamides [51]. The presented results indicate that toxicologically harmless and biodegradable alkyl xylosides, can be considered as an alternative for synthetic commercial surfactants.

### Emulsion and Foam Stability

In order to investigate the surface active properties of these compounds two parameters were chosen, the stability of the emulsion and the foam. The method for measurements of emulsifying capacity of the surfactant water solutions is based on the assumption that the higher OD_600_ is, the more dispersed the emulsion is. Moreover, the lower the absorbance decrease rate is, the more stable the emulsion is. The obtained results are presented in Table 2.

**Table 2:** Emulsification properties of the tested surfactants described by initial OD_600_ and OD_600_ decrease rate values

	Surfactant concentration							
	10 mg L^−1^		50 mg L^−1^		100 mg L^−1^		250 mg L^−1^	
	Initial OD_600_ [−]	OD_600_ decrease rate [min^−1^]	Initial OD_600_ [−]	OD_600_ decrease rate [min^−1^]	Initial OD_600_ [−]	OD_600_ decrease rate [min^−1^]	Initial OD_600_ [−]	OD_600_ decrease rate [min^−1^]
C_8_X	1.02 ± 0.05	0.025 ± 0.001	2.08 ± 0.10	0.021 ± 0.002	2.15 ± 0.11	0.016 ± 0.002	2.96 ± 0.15	0.008 ± 0.001
C_9_X	1.68 ± 0.08	0.032 ± 0.002	2.65 ± 0.13	0.015 ± 0.001	2.58 ± 0.13	0.013 ± 0.002	2.68 ± 0.13	0.013 ± 0.002
C_10_X	2.23 ± 0.11	0.032 ± 0.002	2.84 ± 0.14	0.026 ± 0.005	2.86 ± 0.14	0.023 ± 0.003	2.88 ± 0.14	0.017 ± 0.002
C_12_X	1.98 ± 0.10	0.033 ± 0.002	2.66 ± 0.13	0.016 ± 0.002	2.89 ± 0.14	0.013 ± 0.001	2.77 ± 0.14	0.018 ± 0.002

C_8_X, octyl d-xyloside; C_9_X, nonyl d-xyloside; C_10_X, decyl d-xyloside; C_12_X, dodecyl d-xyloside

The emulsification properties increased with the increased alkyl chain length, obtained maximum for C_10_X, and dropped slightly for C_12_X. However, the difference in dispersion ratio is more significant at lower concentration. What is more, emulsion decrease, measured by OD_600_ decrease rate, declined with the rising surfactant concentration for all tested compounds.

Emulsifiability of alkyl β-d-galactopyranosides, from C_6_ to C_9_, was investigated by Chen *et al*. [54]. They noticed the increase of height of emulsion level with an increasing number of carbons. Alkyl glucopyranosides were also studied by Niraula *et al*. [55] with similar conclusions.

The results of foam formation and its stability for tested alkyl xylosides are summarized in Table 3. For the concentration 10 mg L^−1^ the measurements were also conducted, however, in the case of each surfactant, no stable foam was observed. The increase of foam layer height with the increasing alkyl xylosides concentration was observed for all compounds except for decyl xyloside. For this surfactant the highest foam was generated at the concentration 50 mg L^−1^ (initial height 60.2 mm). Such phenomena may be correlated with a relative high minimum surface occupied by a surfactant molecule, 4.68 × 10^−19^ m^2^ and a low surface excess at the saturated interface, 3.55 × 10^6^ mol m^−2^ (Table 1). These parameters indicate that, among the tested surfactants, C_10_X requires the lowest number of molecules necessary to the saturate the interfacial surface. Moreover, the longer alkyl chain yielded the more stable and more dispersed emulsion. The only significant exception is low foam stability for C_12_X. This may be caused by the more hydrophobic properties of this surfactant, which influenced foam bubble formation.

**Table 3:** The foam measurements for tested surfactants

	Surfactant concentration					
	50 mg L^−1^		100 mg L^−1^		250 mg L^−1^	
	H_0_ [mm]	H_5_ [mm]	H_0_ [mm]	H_5_ [mm]	H_0_ [mm]	H_5_ [mm]
C_8_X	9.1 ± 0.5	1.2 ± 0.2	9.8 ± 0.3	5.1 ± 0.2	10.2 ± 0.3	9.2 ± 0.3
C_9_X	14.8 ± 0.8	6.3 ± 0.3	16.7 ± 0.3	11.8 ± 0.3	19.9 ± 0.4	14.1 ± 0.3
C_10_X	43.2 ± 1.2	35.1 ± 0.5	60.2 ± 0.4	53.1 ± 0.3	39.8 ± 0.4	35.2 ± 0.4
C_12_X	15.9 ± 0.8	1.1 ± 0.2	40.1 ± 0.4	2.2 ± 0.4	60.1 ± 0.5	45.1 ± 0.5

H_0_ and H_5_ represent the height of the foam layer in a cylinder, when the foam generation was stopped and 5 min after then

C_8_X, octyl d-xyloside; C_9_X, nonyl d-xyloside; C_10_X, decyl d-xyloside; C_12_X, dodecyl d-xyloside

The obtained results clearly illustrate the influence of the alkyl chain length on the surfactant foaming properties. Such conclusions were found in several publications [56–58]. Badache *et al*. [21] noticed that a longer alkyl chain of the surfactant is connected with the increasing foamability. Moreover, Koeltzow and Urefer [59] compared the foam amount generated in solutions of alkyl maltopyranosides and maltotriopyranosides with alkyl chain containing from 8 to 18 carbon atoms. They found out that the highest foam level at 1 g L^−1^ was found for a tridecyl chain. Chen *et al*. [54] observed also a maximum in the function between alkyl chain length and the foaming ability of alkyl β-d-galactopyranosides.

### Toxicity of Surfactants

The toxicity of the tested compounds was determined using the MTT assay, indicating the cell metabolic activity. All results were compared with the reference sample without any surfactant, value of which was set as 100%.

**Fig. 2:**
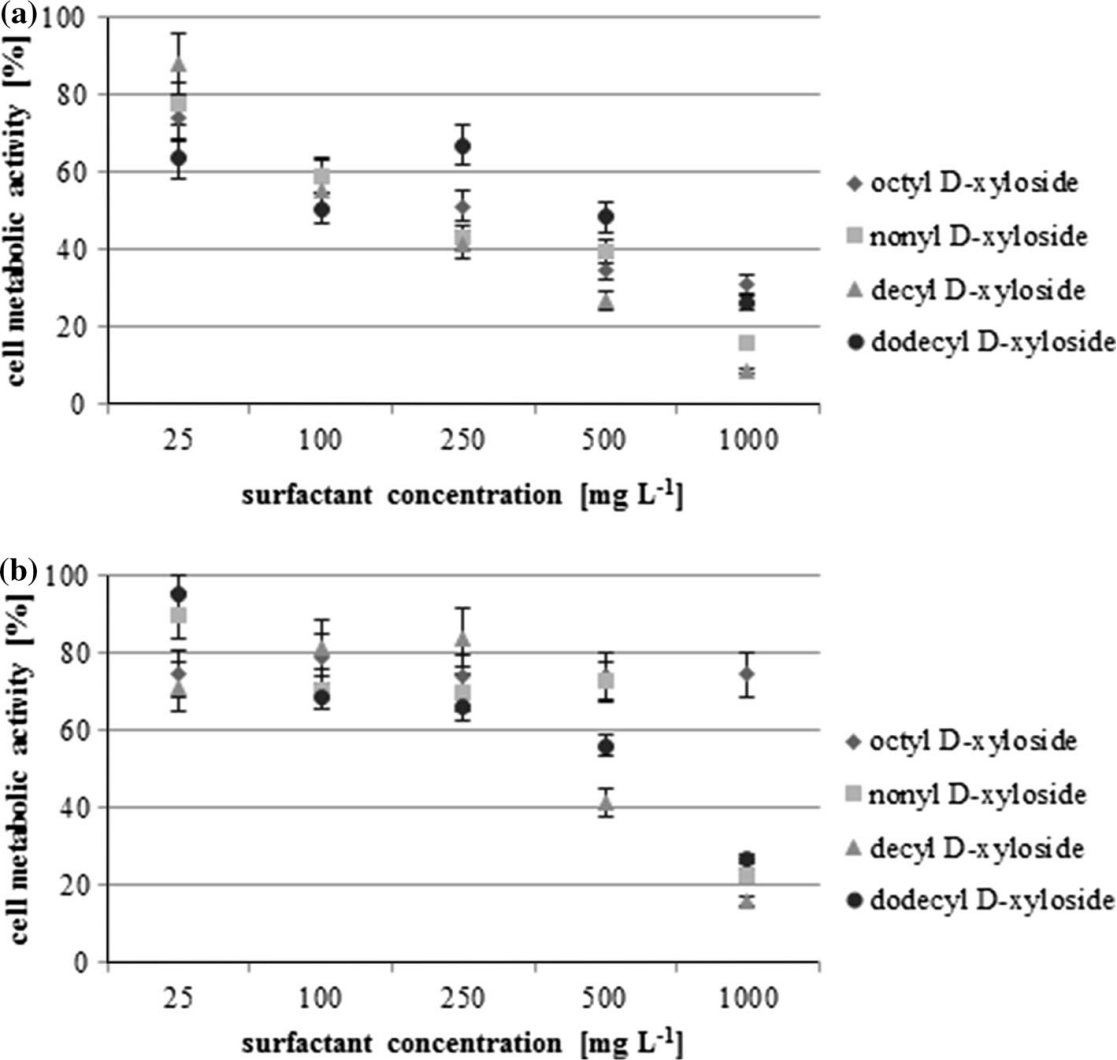
Toxicity of tested surfactants represented by relative cell metabolic activity measured using the MTT assay, the values for: **a** *Pseudomonas fluorescens* ATCC 14700, **b** *Pseudomonas* sp. KG1

The *P. fluorescens* strain was more sensitive than the other strain to the presence of these surfactants (Fig. 2a). The 50% decrease in the cell metabolic activity was observed for C_8_X and C_10_X at a concentration of 250 mg L^−1^. At the same concentration the metabolic activity of C_9_X and C_12_X was 55 and 65% respectively. At the highest tested concentration, 1000 mg L^−1^, the metabolic activity did not exceed 35% for C_8_X and 10% for C_10_X.

In the case of *Pseudomonas* sp. KG1 (Fig. 2b), the cell metabolic activity in the presence of C_8_X did not fall below 70% even at the highest concentration. This level was not exceeded for C_9_X at 500 mg L^−1^, and at 250 mg L^−1^ for two other compounds. The metabolic activity at 1000 mg L^−1^ for C_9_X, C_10_X and C_12_X was lower than 30%.

In the literature there are practically no studies about toxicity of alkyl xylosides. Xu *et al*. [60] studied cytotoxicity of alkyl β-d-xylopyranosides in mammalian cells, but no impact on bacteria cells was examined. However, the obtained results correspond well with those obtained by Jurado *et al*. [61] for alkyl polyglucosides. Their results indicated that the toxicity increased as the CMC value decreased, which occurs, when the alkyl chain is longer. Such correlation was also noticed in the case of the toxicity of alkyl polyglucosides against other microorganisms, e.g. *Vibrio fischeri* or *Daphnia magna* [62]. Sahariah *et al*. [63] studied the antibacterial activity of a different group of compounds, *N*,*N*-dialkyl chitosan derivatives, against several Gram-positive strains. They also found out, that the toxicity is correlated with the length of the alkyl chain. However, the order was dependent on the bacterial strain.

### Cell Surface Modifications

**Fig. 3:**
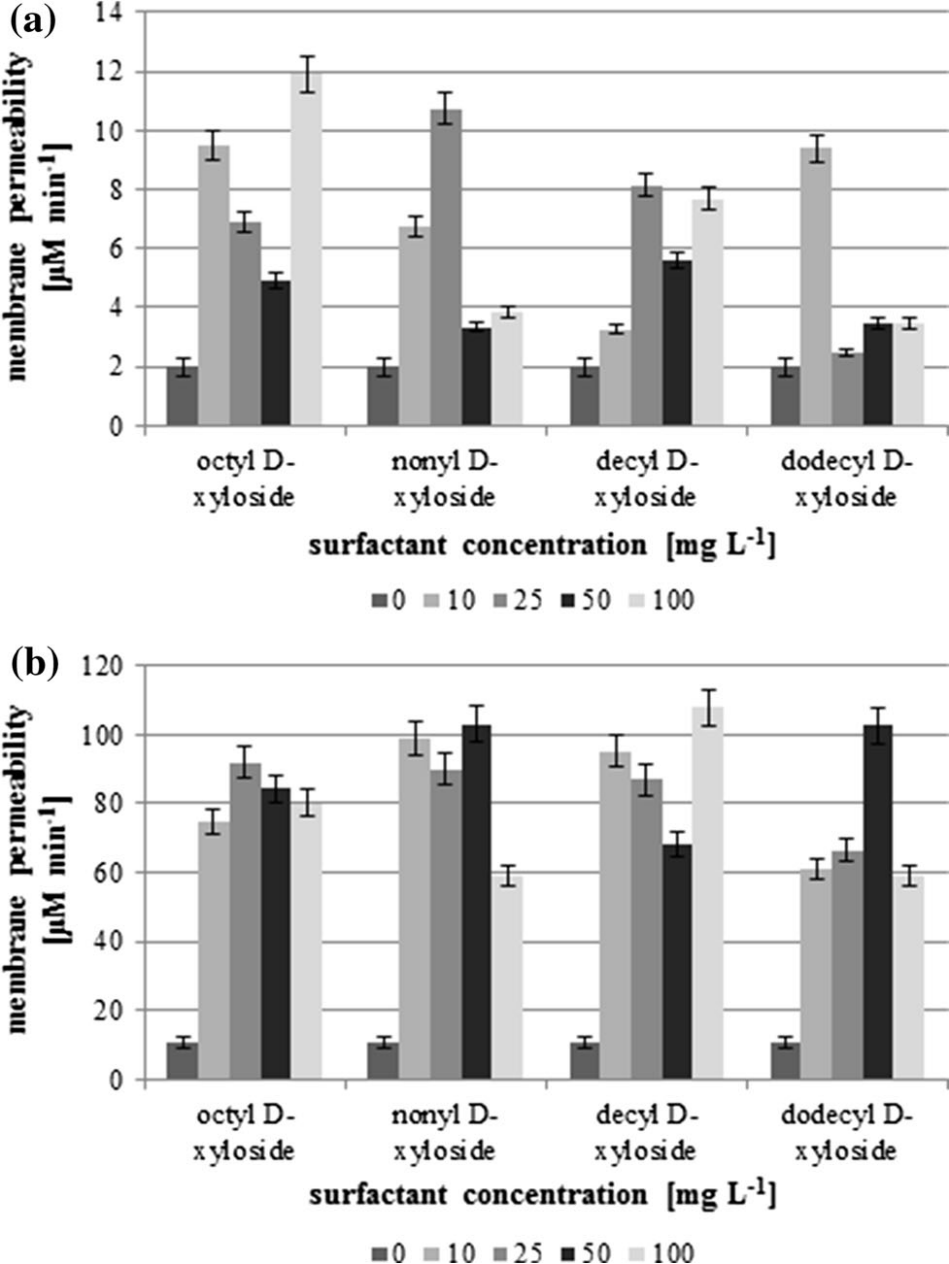
Inner cell membrane permeability changes in the presence of tested alkyl xylosides, **a** *Pseudomonas fluorescens* ATCC 14700, **b** *Pseudomonas* sp. KG1

In order to investigate the impact of the surface active compounds on bacteria cells the cell surface hydrophobicity as well as cell membrane permeability can be considered as sensitive and valuable parameters. The changes of a cell inner membrane in the presence of the surfactants are presented in Fig. 3. What is important, the both strains from the same genera, possessed very different initial values of the membrane permeability, 2 and 12 μM min^−1^, for *Pseudomonas fluorescens* and *Pseudomonas* sp. KG1, respectively. Although, the values differ about ten times between the used strains, in both cases the addition of the tested compounds results in an increase in membrane permeability. However, no correlation between the surfactant concentration and the membrane permeability was found. For *P. fluorescens*, the highest value was observed at 100 mg L^−1^ (C_8_X), 25 mg L^−1^ (C_9_X and C_10_X) or 10 mg L^−1^ (C_12_X). In the case of the second strain, *Pseudomonas* sp. KG1, the rise of cell permeability is more significant, and even at concentration 10 mg L^−1^ the permeability increases over six (C_12_X), seven (C_8_X) or nine (C_9_X and C_12_X) times.

**Fig. 4:**
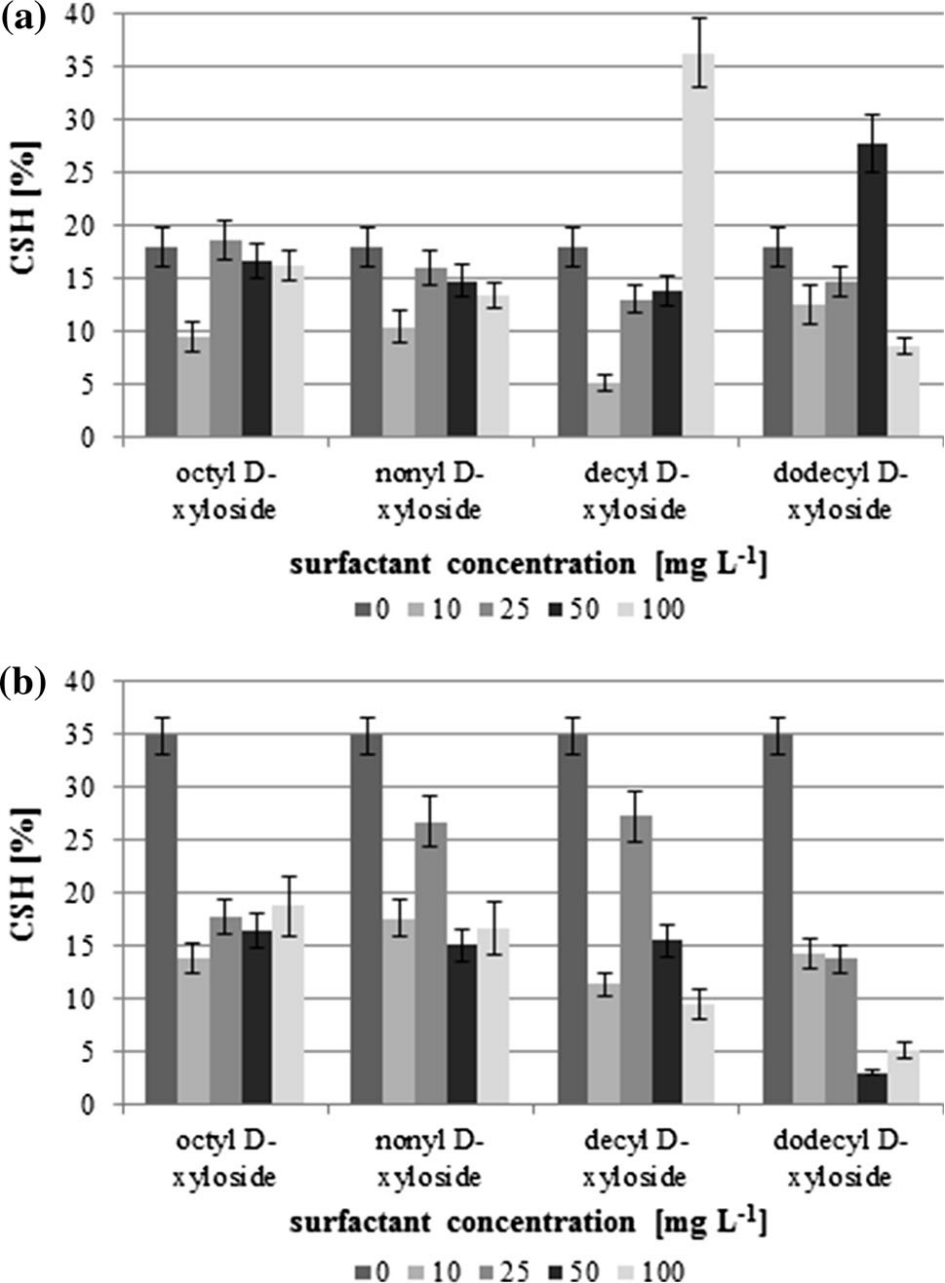
Cell surface hydrophobicity (CSH) **a** *Pseudomonas fluorescens* ATCC 14700, **b** *Pseudomonas* sp. KG1

Analyzing the changes of the cell surface hydrophobicity (CSH) the different strains' response on the presence of the surfactants can be observed. The cells of the reference strain, *P. fluorescens*, were hydrophilic (CSH < 35%) and did not change with rising surfactant concentration (Fig. 4a). More significant cell surface properties modifications were observed in several cases only. The increase up to 36 and 28% was noticed for C_10_X (100 mg L^−1^) and C_12_X (50 mg L^−1^), respectively. The noticeable drop in CSH was observed at 10 mg L^−1^, especially for C_10_X. The response of the *Pseudomonas* sp. KG1 strain cells was different (Fig. 4b). In all cases the significant decrease of CSH was noticed. The initial CSH was at the level 35% and the values dropped even below 5% for C_12_X at concentrations higher than 50 mg L^−1^. Comparing both analyzed parameters, it can be noticed, that, in general, an increase of the cell surface hydrophobicity is accompanied by a decrease of the cell membrane permeability.

The compounds with surface activity are considered as an important modifier of a cell membrane structure and its permeability [64]. Tobe *et al*. [65] observed that methyl ester ethoxylates increase the permeability of the *Escherichia coli* cell membrane. On the other hand, Fuchedzhieva *et al*. [66] noticed that benzene sulfonates (LAS) with linear alkyl chain did not affect the microbial cell permeability of *Pseudomonas* sp. PS-17. It could suggest that the hydrophilic carbohydrate headgroup in alkyl xylosides can play an important role in membrane permeability changes.

The several studies conducted by different researchers suggest that surfactants can be one of the agents influencing cell surface hydrophobicity [33, 41, 67]. The carbohydrate-based surfactants with longer alkyl chains, like alkyl polyglucosides, can affect bacterial surface properties as well. However, the simple dependence between the alkyl chain length or surfactant concentration was not observed [45].

## Conclusions

In summary, we have observed that the tested long-chain alkyl xylosides showed significant surface active properties. They showed considerable ability for surface tension reduction, even at relatively low concentrations. The direct correlation between the number of carbon atoms in the alkyl chain and logarithm of the critical micelle concentration of the surfactants was observed. The analyzed compounds showed also good emulsifying and foaming properties. All tested compounds did not show any toxic influence on two *Pseudomonas* strains at concentrations below 25 mg L^−1^. The surfactants influence both the cell inner membrane permeability and cell surface hydrophobicity. However, the character of the modification depends strongly on the bacteria strain, alkyl chain length, as well as on the surfactant concentration. The studied long-chain alkyl xylosides have potential as environmentally friendly surfactants due to their interesting physico-chemical and eco-toxicological profiles.
